# Brief interventions for drink-driver offenders in Slovenia

**DOI:** 10.1186/1940-0640-7-S1-A32

**Published:** 2012-10-09

**Authors:** Marko Kolšek

**Affiliations:** 1 Department of Family Medicine, University of Ljubljana, Ljubljana, Slovenia

## 

Drunk driving remains a problem in Slovenia despite the 2008 Law on Road Safety, which enables the police to retain intoxicated drivers for 6-12 hours who have a blood alcohol level of more than 1.5 g/kg. Every night, approximately 20 drivers pass a night at the police station (the Slovenian population is 2 million). To reduce drunk driving, an updated Law on Road Safety was approved in December 2010, which introduced higher fines and extra penalty points for drunk driving. It also offered a provision for drunk drivers to receive medical examinations and counseling from their family doctors. Short courses and printed materials for Slovenian family physicians were prepared and disseminated in the spring of 2011 to introduce them to basic principles of brief intervention. In October 2011, the examination and counseling program was launched, and data collection for a study to assess results is underway.

